# Effect of spatial constraints on Hardy-Weinberg equilibrium

**DOI:** 10.1038/srep19297

**Published:** 2016-01-14

**Authors:** Yi-Shin Chen, Yi-Cheng Su, Wei Pan

**Affiliations:** 1 Institute of Information Systems and Applications, and Department of Computer Science, National Tsing-Hua University, 101, Sec. 2, Kuang-Fu Rd., Hisn-Chu 300, Taiwan; 2 Department of Physics, National Dong Hwa University, 1, Sec. 2, Da-Hsueh Rd., Hua-Lien 974, Taiwan; 3 Department of Physics, National Chung Cheng University, 168, Sec. 1, University Rd., Chia-Yi 621, Taiwan

## Abstract

Panmixia is a key issue in maintaining genetic diversity, which facilitates evolutionary potential during environmental changes. Additionally, conservation biologists suggest the importance of avoiding small or subdivided populations, which are prone to losing genetic diversity. In this paper, computer simulations were performed to the genetic drift of neutral alleles in random mating populations with or without spatial constraints by randomly choosing a mate among the closest neighbours. The results demonstrated that the number of generations required for the neutral allele to become homozygous (T_*h*_) varied proportionally to the population size and also strongly correlated with spatial constraints. The average T_*h*_ for populations of the same size with spatial constraints was approximately one-and-a-half times longer than without constraints. With spatial constraints, homozygous population clusters formed, which reduced local diversity but preserved global diversity. Therefore, panmixia might be harmful in preserving the genetic diversity of an entire population. The results also suggested that the gene flow or gene exchange among the subdivided populations must be carefully processed to restrict diseases transmission or death during transportation and to monitor the genetic diversity. The application of this concept to similar systems, such as information transfer among peers, is also discussed.

Maintaining genetic diversity is a key issue in conservation biology. In evolutionary theory, genetic drift is a driving force that is as critical as natural selection and changes the gene ratio of a population in a random manner. In an idealised population that follows Hardy-Weinberg conditions (HW)–(i) sexual reproduction with discrete generations; (ii) random mating; (iii) absent selection, mutation, and migration; and (iv) a sufficiently large population size—the gene ratio of a trait persists for many generations, as described by the Hardy-Weinberg equilibrium (HWE)^1^,^2^,^3^,^4^,^5^,^6^. The HWE is often utilised to determine whether genetic diversity is lost^7^,^8^,^9^,^10^,^11^,^12^,^13^,^14^. In the absence of selection, keeping the population panmictic is important to achieve the HWE. In addition to random mating, which involves choosing a mate despite the genes, panmixia indicates that every other individual in the population is a potential mate. In other words, in a panmictic population, each individual has a chance to become a mate of any other individual. Additionally, population genetics theories are mainly based on expected values in statistics, resulting from panmixia, i.e., the statistical results are based on the assumption of a panmictic population, in which all individuals have the potential to become a mate of all other individuals. That is, there is no restriction on gene exchange among individuals in a population, and each individual has the opportunity to exchange their genes with any other individual in the population. Therefore, the expected values based on probability theory can be applied in population genetics. Nevertheless, whether the expected values determined using probability theory are in agreement with the experimental data is based on randomisation and the law of large numbers. Therefore, panmixia and the population size are important issues in the development of population genetics theories.

Considering the alleles of a locus in a diploid panmictic population with a population size of *N*, the changes in the allelic frequency of the entire population resemble a one-dimensional random walk with two ends of completely homozygous constitutions. That is, at each generation period, the random walker—the allelic frequency—has the opportunity to randomly move 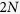 steps on a line with one end being total *AA* and the other end being total *aa*. In this case, there are only two fixed points in the process, and all individuals with a genotype of either *AA* or *aa*, constitute the two absorbing states. When the allelic frequency of *A* becomes 1 (or 0), the allelic frequency ceases to change unless the HW are violated, such as by mutation or migration. An idealised population may deviate from the HWE through genetic drift.

Population sizes must be relatively large to overcome the higher inbreeding probability in small populations, e.g., island populations or subdivided populations^1^,^11^,^15^,^16^,^17^. Previous studies have shown that several small populations (SS) are better than a single large population (SL) with an equivalent total population size in maintaining genetic diversity^18^,^19^,^20^,^21^. A better way of mediating the argument between SS and SL is to include SS and the translocation of some individuals among the SS to avoid inbreeding, i.e., to make the subpopulations resemble a united panmictic population as much as possible. The reason for not translocating animals at a high frequency is the risk of disease transmission among the SS populations^20^. Nevertheless, the changes in allelic frequency because of panmixia has not been discussed and panmixia is usually considered as a requirement for maintaining the genetic diversity. Regarding the issue of population size, experimental and theoretical studies have shown that SS populations maintain the genetic diversity for the entire population but lose local diversity. In contrast, SL populations maintain diversity for the entire population but lose local diversity^18^,^20^. Nevertheless, even in an infinitely large population, the probability of a gamete spreading depends heavily on spatial constraints and decreases with distance. Thus, finding opportunities to obtain a mate far from where one lives can increase the genetic diversity of one’s offspring. For example, female blue tits typically copulate with non-neighbouring males to increase the heterozygosity of their offspring^22^.

According to the Wahlund effect, subdivided populations can become homozygous in the forms *AA* or *aa*, and as a result, the entire population may maintain a near-constant 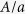 ratio^23^. In other words, it is possible that the alleles in certain small populations are *AA*, whereas others can be *aa* in a SL population. Is panmixia required in maintaining genetic diversity? In this paper, we utilised a computer simulation to elucidate the effects of spatial constraints and population size on genetic drift and discuss the relationship between panmixia and population size. The results reveal that HWE requires more than HW. Under HW, panmixia is harmful to the maintenance of genetic diversity. Thus, selection, mutation, and migration might play significant roles in maintaining genetic diversity.

## Results

The alleles in the entire population inevitably became homozygous at generation 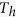 regardless of the population size. The average 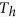, T_*ave*_ is proportional to the population size given the same spatial constraint. Moreover, at the same population size, the T_*ave*_ for the simulation with constraints was larger than that for the simulation without constraints. Thus, spatial constraints increase the T_*ave*_ and maintain gene diversity. In other words, panmixia accelerates the genetic extinction process. Additionally, in the early stages, clusters of homozygous populations were formed, and heterozygous individuals decreased relatively drastically in the G_4_, G_6_, and G_8_ simulations than in the G_*nf*_ simulations, suggesting that local homozygous population clusters are critical for maintaining genetic diversity.

### Allelic Frequency as a Random walk

**Figure 1:**
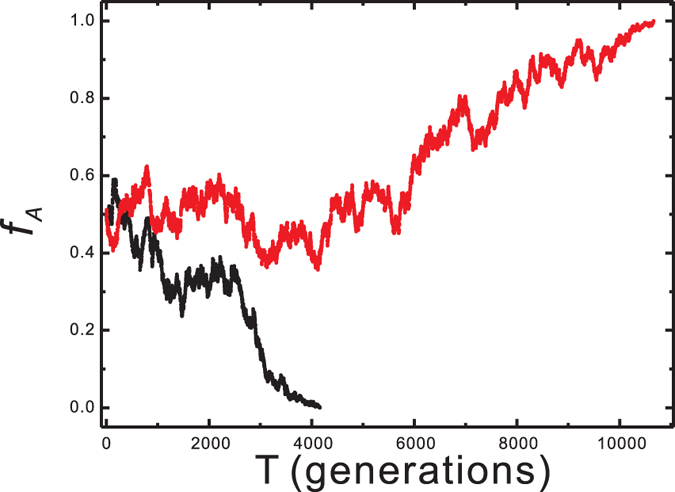
The allelic frequency changes in the simulation. The allelic frequency, *f*_*A*_, is plotted as a function of the generation in G_*nf*_ with a population size of 2,500. The initial value of the allelic frequency is approximately 0.5 and fluctuates in the following generations. The simulation is terminated when the allelic frequency is fixed at 1 or 0.

Because of the probabilistic nature of mate selection and gamete formation, 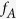 changes from generation to generation in a random manner, begining near 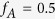 and drifting toward one of the homozygous terminals, 1 or 0, as illustrated in Fig. 1. The genetic drift of a locus in a population as a random walk has been demonstrated. The terminals of 1 and 0 are the two absorbing states of the random walk. When 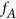 arrives at one of these two points, no more changes in 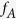 occur. The evolution of the alleles ceases if the conditions do not change.

**Figure 2:**
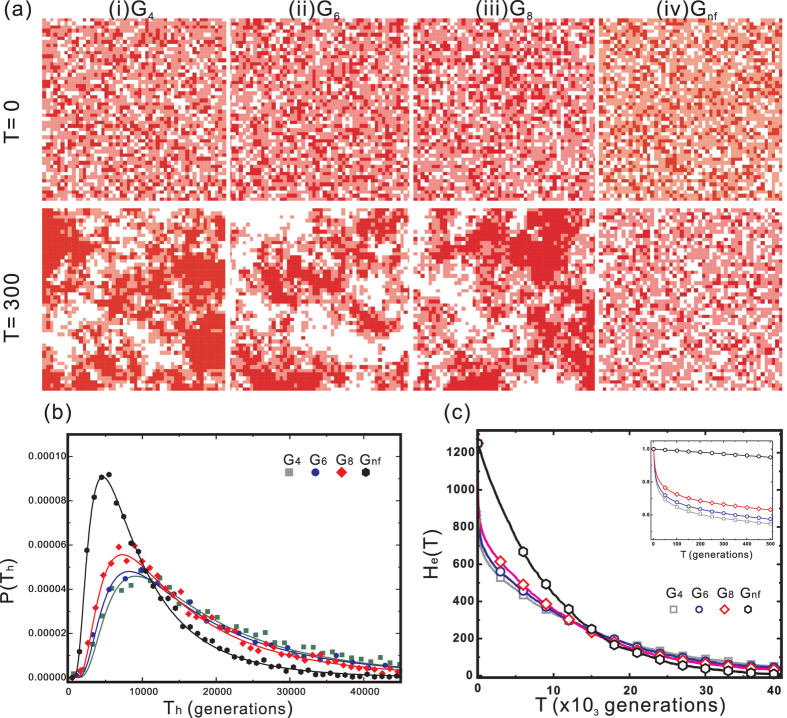
(**a**) Spatial distribution of genotypes in the population size of 2,500 (50 × 50). (**b**) Probability distribution of T_*h*_ using the population of 2,500 as an example. The curves show the fitted inverse Gaussian distributions. These fitted curves are normalised by setting the area under the curve as 1. (**c**) Heterozygosity as a function of the generations (*T*) of a population size of 2,500. The heterozygous populations eventually vanished despite the geographic constraints. In G_*nf*_, the heterozygosity decreased slower than in G_4_, G_6_, and G_8_ in the first 10,000 generations, whereas it was less than in G_*nf*_ after the 15,000^*th*^ generation. The inset illustrates the heterozygosity for first 500 generations.

Regardless of the population size, T_*h*_ as a function of generations exhibits an inverse Gaussian distribution, which describes a first-passage time distribution of a random motion with drift^24^,^25^,^26^. Thus, our simulations have a random nature driven by the absorbing states. Examples for a population of 2,500 are depicted in Fig. 2(b). For other populations, see supplementary information. The peaks of the curves are arranged in the order of G_4_ > G_6_ > G_8_ > G_*nf*_, revealing that spatial constraints lengthen the T_*h*_ that maintains HWE. Because T_*h*_ is the time for which the population maintains the potential to evolve, panmixia might hinder maintaining the evolutionary potential of a population.

### Effects of Spatial Constraints on T_*ave*_

**Figure 3:**
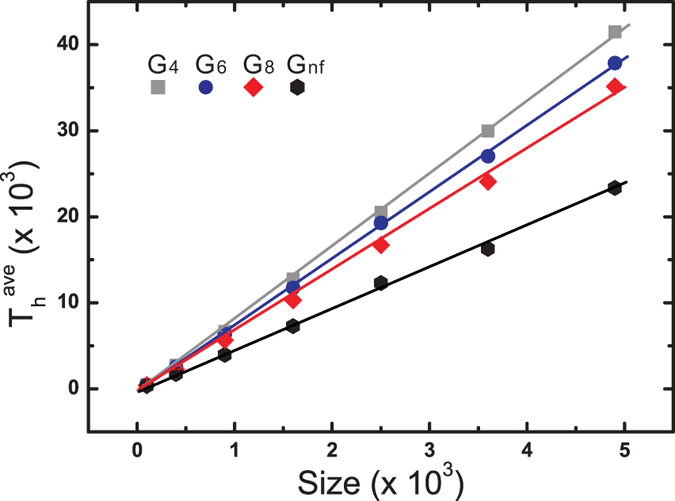
Effects of population size and spatial constraints on the corresponding survival times. For all neighbouring correlations, T_*ave*_ is proportional to the population size for all simulation sets. The slopes (T_*ave*_/size) were 8.44, 7.72, 6.96, and 4.73 for G_4_, G_6_, G_8_, and G_*nf*_, respectively. For the same population size, the T_*ave*_ values for different spatial constraints were ranked in the following order: 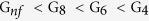. For the same population size, the non-constraint population, G_*nf*_, approached homozygosity faster than the populations with spatial constraints.

T_*ave*_ approximately indicates the time period in which the population possesses evolutionary potential, which is proportional to the population size for the same spatial constraints, as shown in Fig. 3. In other words, larger population size have longer convergence times. The T_*ave*_ values for various spatial constraints applied to the same population size shows that T_*ave*_(*G*_*nf*_) < T_*ave*_(*G*_8_) < T_*ave*_(*G*_6_) < T_*ave*_(*G*_4_), indicating that the spatial constraint is beneficial to maintaining a population’s genetic diversity. In contrast, the panmictic population, G_*nf*_, exhibits reduced genetic diversity for populations with the same size. The slopes (T_*ave*_/size) were 8.44, 7.72, 6.96, and 4.73 for G_4_, G_6_, G_8_, and G_*nf*_, respectively. The population size required to maintain the same T_*ave*_ is inversely proportional to the slope. Compared with the panmictic populations, G_*nf*_, the population sizes with the same T_*ave*_ are 0.56, 0.62, 0.68, and 1.0 for G_4_, G_6_, G_8_, and G_*nf*_, respectively. These values allow us to estimate the reduced effective population size resulting from the spatial constraints. For instance, in G_*nf*_, 1,000 individuals are required to maintain the genetic diversity for approximately 4,000 generations, whereas only 560, 616, and 682 individuals are required for G_4_, G_6_, and G_8_, respectively. This result suggests that increasing the opportunity to obtain a long-distance mate, such as the previously described case of the blue tits, might not benefit the genetic diversity of the entire population.

### Spatial Distribution

Figure 2(a) presents the spatial distribution of the individual genotypes in the simulation sets of (i) G_4_, (ii) G_6_, (iii) G_8_, and (iv) G_*nf*_ for a population of 2,500. The lattice containing an individual carrying the *AA, Aa*, or *aa* genotype is indicated by red, pink, or white, respectively. In the initial generation, 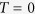, the spatial distribution of the individual genotypes is random for all simulation sets. In the 300^*th*^ generation, i.e., 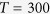, G_4_, G_6_, and G_8_ exhibit the formation of homozygous clusters, whereas the heterozygous individuals distribute at the boundaries of the *AA* and *aa* clusters. This cluster formation occurs because an individual’s gametes can transfer (e.g., carried by pollen) to the nearest neighbours and offspring (e.g., a seed) living in the same lattice. However, no such pattern was observed in G_*nf*_ because the individuals can spread (obtain) the gametes to (from) any other individual in the entire population. In the simulations with other populations, the spatial distributions of the genotypes of the individuals are similar to those of the population of 2,500. Compared with T_*ave*_, as shown in Fig. 3, the coexistence of both *AA* and *aa* homozygous clusters is critical for maintaining genetic diversity. Mating between two parents with alternative genetic constitutions can change the allelic frequency of the entire population, whereas mating between two individuals with the same homozygous alleles cannot. In the simulations with spatial constraints, mating between individuals inside the homozygous cluster does not change the allelic frequency. Only mating between the individuals at the boundary of the homozygous clusters changes the allelic frequency of the entire population. Compared with the populations with spatial constraints, the individuals in a panmictic population have greater opportunity to find a mate with a different genotype, and thus, mating results in allelic changes.

Changes in allelic frequency are equivalent to the movements of a one-dimensional random walk, which drives the walker to one of the two ends of the absorbing states in a reduced number of generations.

### Decrease in Heterozygosity

Heterozygosity decreases dramatically in the early stages of the G_4_, G_6_, and G_8_ simulations but not the G_*nf*_ simulation, as illustrated in the inset of Fig. 2(c). During this period, the order of the heterozygosity is G_4_ < G_6_ < G_8_ < G_*nf*_, whereas *F* is in the reverse order. Thus, spatial constraints lower heterozygosity and increase inbreeding in the early stages. Only the heterozygosity of G_*nf*_ fits the pattern of exponential decay of H_*e*_(*T*) in Eq. 3, which indicates that the spatial constraint causes the mating behaviour to be less random. Compared with the results shown in Fig. 2(a), the formation of homozygous clusters correlates with the decrease in heterozygosity in the early stages of the simulations. After approximately the 15,000^*th*^ generation, the heterozygosity curves are similar for all simulations. In the panmictic population, the decrease in heterozygosity is comprehensive and affects the entire population. However, the decrease of heterozygosity in the population with spatial constraints indicates that the reduction of the heterozygous population in the boundary of the homozygous clusters is associated with the formation of the clusters. In other words, the formation of the homozygous clusters in G_4_, G_6_, or G_8_ protects (one of) the genes from extinction. Therefore, the spatial constraint drives the decrease in heterozygosity in the early stages but maintains the heterozygosity in later stages. As mentioned previously, only the mates at the boundary between two clusters can alter the gene ratio of the entire population. The gene ratio within the cluster does not change from generation to generation because the parents inside the cluster possess identical genotypes.

## Discussion

Our results demonstrate that the genotype of a neutral gene in a population trends toward homozygosity and that one of the alleles (either *A* or *a*) is inevitably eliminated, regardless of the population size. For this reason, the conditions that enable a population to persist at a constant 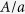 ratio, as described by HWE, must be further elucidated. Our simulations fit HW (i) - sexual reproduction with discrete generations. As indicated by the results that 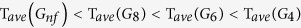, in HW (ii), random mating disrupts the HWE. Moreover, the results of the G_*nf*_ simulation deviate from the HWE more than the others, although its conditions fit HW better than the other conditions in random mating. Thus, the condition of random mating does not support the HWE. Thus, the following question arises: Why does panmixia not support maintaining the HWE in the population?

The probability distribution of T_*h*_ is in an inverse Gaussian form, regardless of the population size and spatial constraints, which indicates that the T_*h*_ distribution of an allele’s extinction process resembles the first-passage time of Brownian motion with drift. The genotype of a gene in the population inevitably approaches the absorbing state at which every individual constitutes a homozygous genotype. This violates the HWE. In the following paragraph, HW (ii), random mating, will be carefully examined.

The allelic frequencies specified by HWE are the expected values based on probability theory, which is assured by the law of large numbers. Nevertheless, fluctuation in the allelic frequency persists during the evolutionary process and drives the system toward steady states. There are two in steady states the allelic frequency of the population: 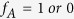. Panmixia causes fluctuations in the allelic frequency of the population because it increases the probability of an individual finding a mate with a different genotype, which causes changes in the allelic frequency. For the fixation of the allelic frequency, one must exclude the probabilistic factors for these two processes. Otherwise, as mentioned previously, the fluctuation drives 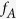 to either 1 or 0. According to HW, there are two probability processes: mate selection and gamete generation; these correspond to steps (ii) and (iii), respectively of our simulation (see Sec. Method). Only the gamete generated by an individual that carries homozygous alleles constitutes a certain allele. For the fixation of the allelic frequency in step (ii), an individual that carries homozygous alleles must find another individual that carries the same homozygous alleles, i.e., 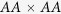 or 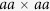, to have offspring with *AA* or *aa*, respectively. In this way, the allelic frequency is certain to be fixed. The mating of 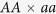 must be compensated by another mating by 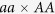 and vice versa. Combinations of a mate with *Aa* encounter a random factor in generating the gamete, which may lead to changes in the allelic frequency. To fix the allelic frequency under the random factors, an increase in 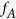 by a mate must be compensated for by an increase in 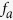 by another mate. The compensation between 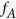 and 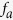 relies on the law of large numbers, which does not guarantee fixation. The drastic decline in heterozygosity in the simulation with spatial constraints, as shown in the inset of Fig. 2(c), indicates that the individual has lower probability of finding a mate carrying a different genotype than in G_*nf*_, such that the 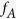 in G_*nf*_ approaches the steady state faster than the simulation with spatial constraints. To fix the allelic frequency, the random factors in step (ii) and (iii) in our simulation must be excluded. However, step (iii) of gamete generation is intrinsically a random process. The fluctuation in allelic frequency induced by this step is unavoidable in our diploid population. The random factors cause 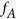 to approach either 1 or 0. In a panmictic population, the driving forces are stronger than those in a non-panmictic population. Therefore, in our simulation, 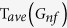 is shorter than those determined for other populations with the same population size. Moreover, the fluctuations can be considered as a one-dimensional random walk with an equal probability of moving a step in the positive or the negative direction. The spatial distribution after a certain period, other than in the HWE, can be modelled by the Wright-Fisher model, which applies probability theory to describe changes in gene frequency and predicts that the alleles of a gene in a haploid (or diploid) population tend toward homozygosity, which leads to the extinction of one of the alleles, reducing genetic variation^16^,^25^,^26^,^27^,^28^,^29^,^30^,^31^,^32^,^33^,^34^,^35^. Our results support the Wright-Fisher model. For details see supplementary information.

The spatial constraints allow the gene to spread only to nearby areas, which has often been considered to increase the inbreeding rate and decrease genetic diversity. Biological conservationists suggest avoiding such a situation, particularly for small populations or for those in island environments^11^,^36^,^37^. However, in our study, the T_*ave*_ of the panmictic population (G_*nf*_) is shorter than that of the populations with spatial constraints (G_8_, G_6_, or G_8_). In other words, the spatial constraints result in heterozygosity loss and enhance the inbreeding rate but postpone the allele’s extinction. The appearance of clusters of homozygous individuals under spatial constraints increases the local homozygosity in the early stages and converges to *aa* or *AA* clusters with an equal probability, thereby maintaining the 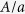 ratio of the entire population. Nevertheless, the equilibrium is unstable and is driven toward an 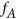 of either 1 or 0 by the fluctuations resulting from gamete generation and random mating. In HW (iv), a sufficiently large population size only postpones the allele’s extinction, unless the population is infinitely large. Nevertheless, previous studies have demonstrated that HWE exists in real populations^38^. Therefore, migration, selection, mutation, or combinations thereof in HW (iii) play critical roles in maintaining the HWE. Migration changes the population size and may also alter the 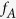. Immigration increases the population size, which prolongs T_*h*_, whereas emigration decreases the population size and enhances the genetic drift that leads the population away from the HWE. Nevertheless, emigration may also cause the population to become divided into multiple small populations, which reduces the T_*h*_ of the subdivided populations but enhances the persistence of both alleles. The size of the subdivided population should be larger than that of the effective population to resist certain diseases caused by inbreeding^17^. Migration affects not only the population size but also the contacts between individuals that transcend spatial restrictions. This effect might be similar to that of long-distance mating as G_*nf*_ in our simulation.

Selection that favours heterozygous individuals can preserve both *A* and *a*, whereas selection that favours *AA* or *aa* shortens T_*h*_. For example, in malaria-stricken areas, heterozygous people (*Aa*) who carry a normal allele (*A*) and an abnormal allele (*a*) of the haemoglobin gene can survive better than homozygous people^39^. Because both alleles survive under such selection, a patient with sickle cell disease who carries two abnormal alleles (*aa*) does not become extinct by selection in these areas, although such a genetic combination is lethal for the carriers. Furthermore, the preference of the selection is time dependent, i.e., it occasionally favours *AA*, and *aa* can sometimes maintain the HWE. Selection that favours *AA* or *aa* is also geographically dependent. HWE can also hold in the entire population.

The effect of mutation on the population size is complicated and might be harmful, beneficial, or neutral for individuals. Mutation that is harmful or beneficial is regulated by selection. A neutral mutation might not only increase genetic diversity but prevent the entire population from becoming genetically homozygous. We suggest revising the conditions for preserving the allele’s frequency as follows: (i) sexual reproduction with discrete generations; (ii) random mating but not panmixia; (iii) selection favouring heterozygosity or time-location-dependent selection is allowed; (iv) absent mutation; and (v) a large population size.

Drifting toward an allele’s extinction is a natural tendency. Therefore, maintaining the HWE requires the cooperation of the aforementioned forces, thereby enabling the coexistence of both alleles. Whether an SL or SS population with the same total size can maintain genetic diversity is debated. Some experimental work has revealed that SS is better than SL in maintaining genetic diversity. Our results support this conclusion. Nevertheless, to avoid inbreeding, transferring individuals among captive animals is recommended. Panmixia is not a solution to increase genetic diversity. Thus, transfers must be performed carefully to monitor possible disease transmission and changes in genetic diversity.

Suppose that there are two genes (two pairs of alleles, *Aa* and *Bb*) that are genetically independent, located on different chromosomes and have no metabolic correlation. The spatial distributions for the individuals that carry these two genes should form homozygous clusters and distribute independently. If the population encounters a severe infection of a disease that is resisted by gene *b*, the number of individuals that carry *bb* should markedly increase. Because the distributions of 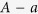 and 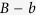 are independent, the population ratio of 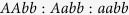, which survive the disease, is approximately the same as that of 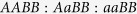 and 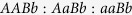, which suffer from the disease. However, the population carrying *BB* or *Bb* is reduced by the disease, which in turn reduces T_*h*_, causing the population to develop a homozygous *AA* (or *aa*) genotype faster than the population that does not contract the disease. An environment (disease) that selects a gene (*b*) might drive another genetically independent gene 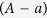 to lose heterozygosity. In other words, the co-evolution of genes does not imply that the genes are genetically dependent, i.e., allocated in the same chromosome or metabolically correlated. Co-evolution might occur among genes that are completely unrelated simply because they are carried in the same individual. Without natural selection, the genotypes in an individual of a population are distributed as homozygous clusters, as shown in Fig. 2(a). The same situation would occur if more alleles had been considered, which would have led to clusters of different combinations of homozygous alleles.

The loss of genetic diversity in a random mating population occurs more rapidly than that in a population with spatial constraints. During the loss process in G_*nf*_, no homozygous clusters are formed, which implies that long-distance genetic exchanges diminish the differences between clusters caused by spatial segregation. The evolution of *Astyanax* cavefish is a relevant example. *Astyanax* cavefish have completely blind, cave-dwelling forms, in addition to surface-dwelling forms with strong vision. The two forms can interbreed and produce fertile hybrids^40^,^41^. The first-generation offspring of the two forms are phenotypically intermediate, and the second-generation offspring range phenotypically from almost completely blind to those with almost normal vision^42^,^43^,^44^, implying that surface-dwelling and cave-dwelling fish have different forms of homozygous alleles that control vision. This supports our results that spatial constraints lead to the formation of homozygous clusters that preserve the genetic diversity of the entire species. The loss of the visual ability of cave-dwelling fish might be attributed to insufficient food supply in the cave surroundings, such that the cave-dwelling fish are superior in energy conservation compared with the surface-dwelling fish^45^,^46^. In other words, the loss of vision benefits evolution in terms of saving energy. However, in a dark cave, the blind, cave-dwelling fish possess no advantage, except for energy conservation. In addition to this selection, the natural tendency to become homozygous can facilitate understanding the occurrence of blind fish.

The concept of evolution in genetics can be extended to evolution in ecology and linguistics, as proposed by Blythe and McKane^35^, and to memes (i.e., cultural ideas and behaviours), as proposed by Dawkins^47^. Dialects, variants, and speech in language evolution correspond to genes, alleles, and populations in genetics^35^. In sociolinguistic research, regional dialect levelling, a process that reduces the differences among regional varieties, is enhanced by vertical social and geographical mobility that transcends close-knit social networks, which maintain local linguistics^48^,^49^,^50^,^51^. Frequent long-range genetic exchanges reduce the global diversity, as shown in G_*nf*_ in our study, similar to the process of regional dialect levelling.

Our model also resembles the information influence among peers under spatial restrictions. For example, a bisexual individual on a square lattice in the G_4_, G_6_, and G_8_ simulations is equivalent to a peer on a node in a homogeneous network with degrees of four, six, and eight, respectively. The information carried by the peer resembles the gene carried by the individual. The two alleles of a gene can be considered as two versions of a particular piece of information, such as two spin states in a magnetic material, two opinions regarding an issue, two dialects of a particular language, or votes in an election with two candidates. The genetic configurations of *AA, aa*, and *Aa* are, respectively, analogous to the following: (1) spin up, down, and undetermined in a magnetic material; (2) pro, anti, and neutral opinions regarding an issue; (3) dialect 1, dialect 2, and bilingual speakers of a language; and (4) candidate 1, candidate 2, and neutral preferences in an election, as shown in Table 1. Peers can be influenced and change the carried information according to the neighbouring, interacting peer. In light of such comparisons, the information transfer among peers in a homogeneous network (G_4_, G_6_, G_8_) is retarded, unlike that in a random network (G_*nf*_). The long-distance transfer facilitates the unification of information, such as opinions, dialects, or voting preferences. In the terminology of idea flow, the entire population becomes an “echo chamber” so that no other voice can be heard when the carried information loses diversity. In contrast, the clusters in our simulation become a local echo chamber in which the cluster members perceive a single voice. Nevertheless, other chambers produce different tones. To spread information more quickly, long-distance idea exchange is necessary. Otherwise, the local echo chambers resist such spreading. In our model, the two alleles are equal, but one of them becomes extinct, implying that, without practicing, long-distance idea exchange is not always an optimal way to discuss an issue because the entire population will soon become a large echo chamber that echoes a poor idea. Furthermore, the long-distance exchange made possible by globalisation might enhance domestic diversity but diminish global diversity. Genetic spreading in populations is analogous to ideas spreading in cultures because both perform self-replication and mutation and adapt under selection^47^. The concept that frequent or long-distance exchange of genes or ideas does not necessarily benefit global diversity should be explored in the future.

Losing diversity via information transfer is a natural tendency. For a neutral gene, the genotype tends toward homozygosity, and one of the alleles becomes extinct, regardless of population size. The formation of local homozygous population clusters reduces local diversity but resists the tendency, thereby maintaining global diversity. Our results suggest that diversity in genetics, languages, or information might similarly be lost in exchanges among respective heterogeneous clusters.

## Methods

**Figure 4:**
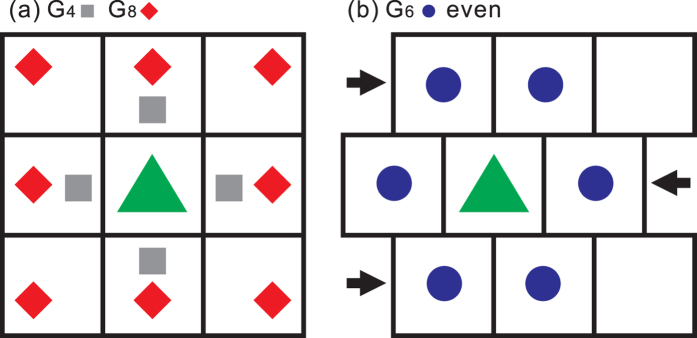
Simulation step (ii), mate selection, in simulations with spatial constraints. In (**a**), an individual (green triangle) located at the centre lattice randomly selected a mate from one of the four neighbours (grey square) for G_4_ or one of the eight neighbours (red diamond) for G_8_. In (**b**), the chessboard was geometrically modified such that the lattices on the even lines shifted to the left by half of a lattice. For G_6_, an individual located in a lattice in the even lines randomly selected a mate from the six neighbours (blue circle).

In the simulations, we considered the genetic drift for a gene in a locus with neutral alleles comprising *A* and *a* in diploid and bisexual populations under the first three HW conditions with spatial constraints. Here, the individuals could randomly locate a mate in nearby areas or from the entire population of different population sizes. The mating is random because the selection of a mate is independent of the genetic constitution. Each individual randomly selected a mate with or without spatial constraints and produced offspring that lived at the same location as the individual, as indicated by the green triangle in Fig. 4. When applying the spatial constraints, the individuals randomly selected one of the four, six, or eight nearest neighbours for the simulation sets, denoted as G_4_, G_6_, or G_8_, respectively. In the simulation set without spatial constraints, denoted as G_*nf*_, the individuals were able to randomly select any other individual as a mate. In this G_*nf*_, the population is panmictic because all individuals are potential mates. The mate-selection process is illustrated in Fig. 4. The program is written in C^++^ programming language. The computer program can be found in Supplementary Dataset.

Without sacrificing the topological correlation, the individuals were allocated to a lattice of chessboards with sizes of 10 × 10, 20 × 20, 30 × 30, 40 × 40, 50 × 50, 60 × 60, and 70 × 70. The periodic boundary condition was applied for individuals that were located at the edges of the chessboard. For example, the nearest four neighbours to the individual located at (1, *i*) on a 10 × 10 chessboard were (1, 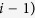, (1, 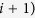, (2, *i*), and (10, *i*). Without losing generality, the results from the population of 50 × 50 were used to demonstrate the spatial and T_*h*_ distributions.

**Figure 5:**
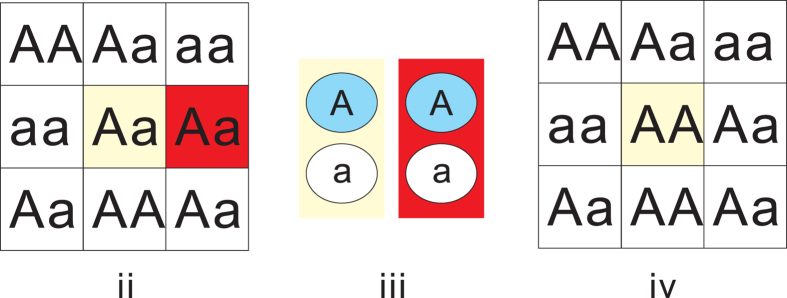
Simulation steps (ii) to (iv). After the first step, initiation, each individual randomly selected a mate from others in the population according to the simulation sets shown in Fig. 4. We take G_4_ as an example. The randomly selected mate is the right-side neighbour (red) in step (**ii**). In step (**iii**), the individual (yellow) and the selected mate (red) randomly provide one of their alleles to form the gametes. The alleles of the gametes provided by the individual and the mate are *A* and *A*, respectively (blue). In step (**iv**), the two gametes combine to form the alleles for the next generation, which is located at the same location as the individual.

The individual and the selected mate donated one gene each to the offspring according to Mendelian rules. The offspring lived at the same lattice as the individual, as indicated by the green triangle in Fig. 4. Initially, the probability of the occurrence of *A* or *a* is set to 0.5, i.e., 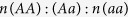 is approximately 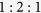. The simulation procedure is divided into four steps: (i) initiation, (ii) mate selection, (iii) gamete generation, and (iv) birth of the next generation. In stage (i), initiation, the program can randomly generate *A* or *a* according to a given probability of *p* and 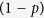, respectively. For an individual, the program generates the probability twice for the alleles of the locus. The overall allelic frequency is approximately *p*, which is 0.5 in this study. In step (ii), mate selection, every individual can randomly take another individual in the population as a mate. The candidate mate is confined by the simulation setup. In the setup with spatial constraints, the candidate mate is selected randomly from the four, six, or eight nearest neighbours, denoted as G_4_, G_6_, or G_8_, respectively; see Fig. 4. In the setup of the panmictic population, denoted as G_*nf*_, the candidate mate is randomly selected from any other individual in the population. In step (iii), gamete generation, each parent randomly takes one of the two alleles with equal probability as the gamete. In step (iv), the birth of the next generation, the alleles provided by both parents are combined to form the gene for the next generation at the same location as the individual. The four stages are repeated for each individual in the population. The steps from (ii) to (iv) are illustrated in Fig. 5. The simulation was terminated when the gene ratio of 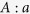 became fixed, that is, the genotypes of all individuals became homozygous, either *aa* or *AA*. For each simulation, the generation at which the population became homozygous was recorded as 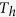.

The average T_*h*_, T_*ave*_, was calculated for each simulation set after running the simulation 10,000 times. Without losing generality, the results for the spatial distribution of the genotypes of the individuals and the time-evolution of 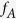 presented in this paper were selected from the simulation of a population size of 2,500.

The allelic frequency, *f* is the portion of the alleles in the entire population as formulated below.


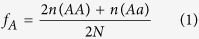



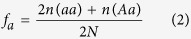


The numbers of individuals with *AA, Aa*, and *aa* alleles are denoted as 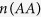, 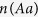, and 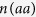, respectively. For simplicity, the allelic frequency is presented in terms of 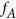, where 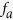 is 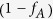.

Heterozygosity is a measure of genetic diversity^17^.


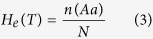



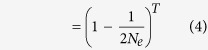



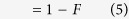


Equation 3 shows the correlation of expected heterozygosity at generation *T*, H_*e*_(*T*) and the effective population size (N_*e*_) for a random mating population, in which *F* is the inbreeding coefficient^11^.

## Additional Information

**How to cite this article**: Chen, Y.-S. *et al*. Effect of spatial constraints on Hardy-Weinberg equilibrium. *Sci. Rep.* **6**, 19297; doi: 10.1038/srep19297 (2016).

## Supplementary Material

Supplementary Information

Supplementary Program 1

Supplementary Program 2

Supplementary Program 3

Supplementary Program 4

Supplementary Program 5

Supplementary Program 6

## Figures and Tables

**Table 1:** Comparison of terms in genes and memes.

configuration	homozygous	homozygous	heterozygous
gene	AA	aa	Aa
magnetization	up	down	uncertain
opinions	support	against	neutral
dialects	dialect 1	dialect 2	bilingual
votes	candidate 1	candidate 2	neutral
