# Supplementary material for: Effect of spatial constraints on Hardy-Weinberg equilibrium
Source: Sci Rep. 2016 Jan 14;6:19297. doi: 10.1038/srep19297 (PMC4725899; doi:10.1038/srep19297)
Supplement: Supplementary Information [file srep19297-s1.pdf]

Effect of spatial constraints on Hardy-Weinberg equilibrium
Supplementary Material

Yi-Shin Chen[1], **Yi-Cheng Su**[2], **and Wei Pan**[3,*]

[1]101, Sec. 2, Kuang-Fu Rd., Institute of Information Systems and Applications, and Department of Computer Science, National Tsing-Hua University, Hisn-Chu 300, Taiwan
[2]1, Sec. 2, Da-Hsueh Rd., Department of Physics, National Dong Hwa University, Hua-Lien 974, Taiwan
[3]168, Sec. 1, University Rd., Department of Physics, National Chung Cheng University, Chia-Yi 621, Taiwan
[*]weipane@gmail.com

ABSTRACT

In this file, we provide (1) probability distribution of T_h for simulation sets, which are fitted by inverse Gaussian distribution with variant parameters, (2) details about Fisher-Wright Model, and (3) Legends for the files in Supplementary Dataset.

Probability distribution of T_h

Probability distribution of T_h for simulation sets of G_4, G_6, G_8, and G_{nf} with populations of 100, 400, 900, 1600, 3600, and 4900 are shown in Fig. 1. The curves show the fitted inverse Gaussian distributions. These fitted curves are normalised by setting the area under the curve as 1.

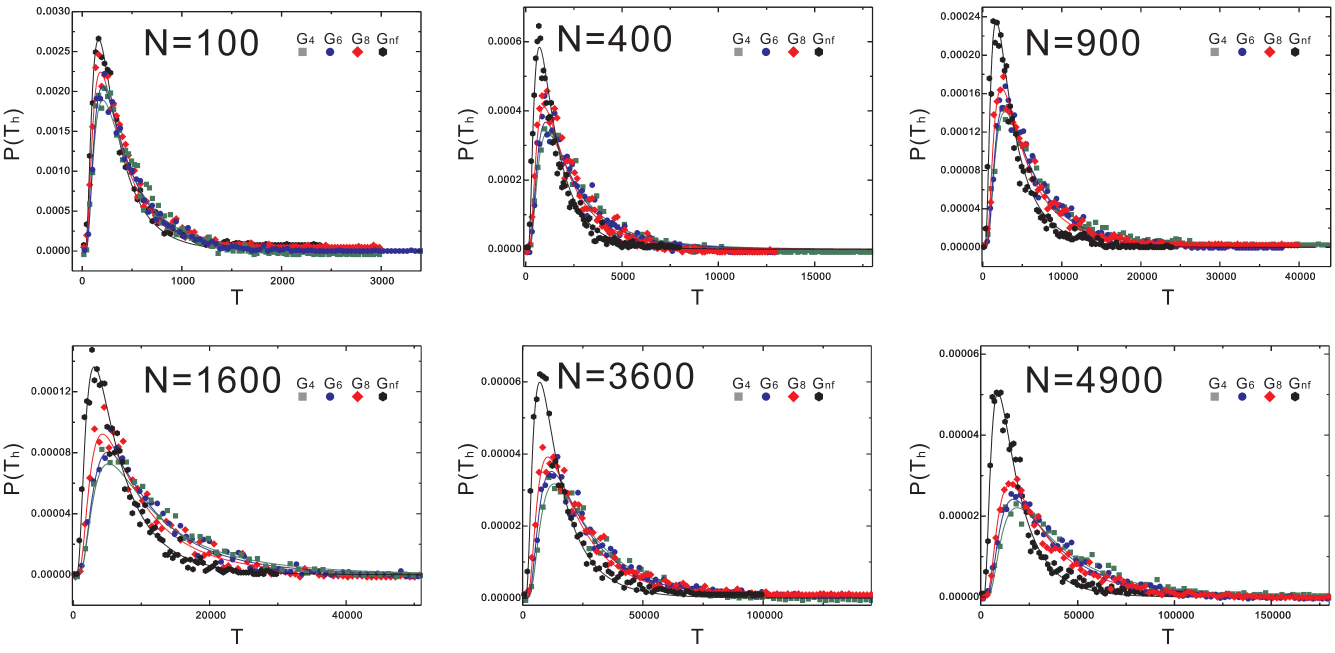

Figure 1. Probability distribution of T_h. The curves show the fitted inverse Gaussian distributions.

Fisher-Wright Model

The process mimics the Fisher-Wright model, which describes the allelic ratio in terms of a transition matrix with an element of transition probability, $q_{i,j}$, whereby the probability for the gene ratio of a haploid population can be shifted from i/N to j/N. This can be extended to a diploid population by replacing N with $2N$. Consider, for example, a population of N individuals as described in the paper. The probability of generating the gametes of A and a is the allelic frequency of f_A and f_a as shown in equation 1, assuming that every gamete has the same probability of combining with another gamete to form the locus of the next generation. The probability that f_A drifts from i to j from generation t to $(t+1)$ is $q_{i,j}$, as shown in equation 1, where C_j^{2N} is the binomial coefficient, $(2N)!/j!(2N-j)!$.

$$q_{i,j} = C_j^{2N} (\frac{i}{2N})^j (1 - \frac{1}{2N})^{2N-j} \tag{1}$$

The matrix $\mathbf{Q}$ is composed of the element of $q_{i,j}$ at row i and column j, respectively. We then define a transition matrix $\mathbf{P}$, which is the transpose of $\mathbf{Q}$:

$$P_{i,j} = Q_{i,j}^T \tag{2}$$

$$= C_i^{2N} (\frac{j}{2N})^i (1 - \frac{1}{2N})^{2N-i} \tag{3}$$

The eigenvalues of this transition matrix $P_{i,j}$ are the same as for $Q_{i,j}$, and they have been solved as shown in equation 4:

$$\lambda_k = \frac{2N!}{(2N-k)!} (\frac{1}{2N})^k \tag{4}$$

Note that both λ_0 and λ_1 are 1, which corresponds to eigenvectors $[1\ 0\ 0\ \ldots]^T$ and $[0\ 0\ \ldots\ 1]^T$, respectively. These two states are the absorbing states corresponding to AA or aa as constituted in every individual in the entire population.

The probability that f_A is equal to a given value m in the tth generation is described by the $(m+1)^{th}$ element, with s_{m+1} in a column vector $\mathbf{S}_t$. The initial state $\mathbf{S}_0$ with an f_A of h is set so that $s_{h+1} = 1$ and all elements are 0. The probability of $f_A = k$ at $(t+1)$, $f_A(t+1) = k$ for the consecutive generation, is presented in a matrix form as shown in equation 5:

$$Prob\{f_A(t+1) = k\} = \sum_j P_{k,j} \times Prob\{f_A(t) = j\} \tag{5}$$

Thus, the state vector at t generation can be written as equation 6:

$$\mathbf{S}_{t+1} = \mathbf{P} \times \mathbf{S}_t \tag{6}$$

$$\mathbf{S}_t = \mathbf{P}^t \times \mathbf{S}_0 \tag{7}$$

$$\mathbf{S}_t = \mathbf{V}\mathbf{D}^t\mathbf{V}^{-1} \times \mathbf{S}_0 \tag{8}$$

This is followed by the diagonalisation of $\mathbf{P}$ to the $\mathbf{VDV}^{-1}$ form, where $\mathbf{V}$ is the eigenvector and $\mathbf{D}$ is composed of eigenvalues at the diagonal terms. However, solving equation 8 is outside the scope of the present paper. The extinction probability at generation t can be obtained by recursive calculation from $\mathbf{S}_0$. That is, the first element, s_t^1 and the last element, s_t^{2N+1} correspond to $f_A(t) = 0$ and 1, respectively.[1,2] Additionally, both s_t^1 and s_t^{2N+1} as functions of t behave as a discrete analog of an Inverse Gaussian distribution. It should be noted that this model can be applied to haploid populations, where the gene number is taken as N . Yet, it cannot be wholly accurately applied to diploid populations. For example, consider a diploid population with $f_A = \frac{2}{2N}$, which contains a allele of 2 and A of $(2N-2)$. There are two possible cases: (a) one individual carries aa and other $(N-1)$ individuals carry AA, and (b) two individuals carry Aa and all other $(N-2)$ individuals carry AA. For case (a), it is not possible for the entire population to become AA in the next generation, whereas the probability of the population becoming homozygous is non-zero in case (b). Nevertheless, the calculation from the Fisher-Wright model does not distinguish between these two cases. That is why we did not use the Fisher-Wright model to describe the simulation in our study.

Supplementary File Legends

The files, Program 1, Program 2, Program 3, Program 4, Program 5, and Program 6 in the Supplementary Dataset correspond to flower.cpp, flower.h, function.cpp, function.h, progmain.cpp, and instructions.pdf. The files are described as below:

flower.cpp: Source code for flower.h.

flower.h: Defines *flower* class, which implements mating progress, and *files* class, which deals with iostream.

function.cpp: Source code for function.h.

function.h: Defines global functions that shared by all files.

progmain.cpp: Main function and program entry. Main function is defined in this source code file. Neighbour type and spatial condition are defined in *tmain*() function. Initialised parameters are also input in *tmain*().

instructions.pdf Instructions for the above files.

It is summarised as shown in Table 1.

File Name	Corresponding File	Short Description
Program 1	flower.cpp	Source code for flower.h
Program 2	flower.h	Definition of *flower* class and *files* class
Program 3	function.cpp	Source code for function.h
Program 4	function.h	Definition of global functions
Program 5	progmain.cpp	Main function and program entry
Program 6	instructions.pdf	Instructions

Table 1. Short description and the corresponding files for the files in Supplementary Dataset.

References

1. Buss, S. R. & Clote, P. Solving the fisher-wright and coalescence markov chain analysis. *Adv. Appl. Prob.* **36**, 1175 – 1197 (2004).

2. Imhof, L. A. & Nowak, M. A. Evolutionary game dynamics in a wright-fisher process. *J. Math. Biol.* **52**, 667–681 (2006).
