# Supplementary material for: Effect of spatial constraints on Hardy-Weinberg equilibrium
Source: Sci Rep. 2016 Jan 14;6:19297. doi: 10.1038/srep19297 (PMC4725899; doi:10.1038/srep19297)
Supplement: Supplementary Program 6 [file srep19297-s7.pdf]

Instructions

I. Overview

This C++ program simulates population genetics that analyzed in the paper with or without spatial constraints.

The simulation procedure is divided into four stages: (i) initiation, (ii) mate selection, (iii) gamete generation and (iv) birth of the next generation.

An $n \times n$ matrix is used to simulate the mating procedure. Each element of the matrix that presented an individual is initialized by a pair of genes (*AA*, *Aa,* or *aa*), which randomly generated from *A* or *a* according to a given probability in stage (i). In stage (ii), (iii) and (iv), each element is randomly mated by one of its 4 neighbours (G_4), 6 neighbours (G_6) or 8 neighbours (G_8) in one loop. The loop stops till all elements constitute the same genes (either *AA* or *aa*). For the simulation without spatial constraint (G_{nf}), an element is randomly mated by any other element. When encounter boundary, a periodic boundary is applied to ensure that the neighbouring condition of each element is the same. In each loop, number of gene *A*, gene *a,* and the distribution are recorded.

Simulation parameters are input manually and results are recorded in local text files.

II. Description of Files

progmain.cpp:

Main function and program entry. Main function is defined in this source code file. Neighbour type and spatial condition are defined in *_tmain()* function. Initialized parameters are also input in *_tmain()*.

flower.h:

Defines *flower* class, which implements mating progress, and *files* class, which deals with iostream.

flower.cpp:

Source code for flower.h.

function.h:
Defines global functions that shared by all files.

function.cpp:
Source code for function.h.

III. Procedure

Input:

1. neighbour type should be predefined in **progrmain.cpp**.
2. after neighbour type is defined, run **progmain.cpp**.
3. In the end, the number of columns and rows, simulation times and random seed are requested to input in terminal.
4. When simulation finishes, the terminal will close automatically.

Output:

The number of generations for each simulation procedure and total time cost are recorded in *generation.txt* file. It will also generate a folder for each simulation named *00x, x* for indexing.

In each simulation folder, there are two text files named *distribution* and *scores* respectively. The file, *distribution* records the constitution of genes for each element in all loop. The file, *scores* records the number of different genes for each loop.

Code Access

All codes are also available on github.
https://github.com/weipane/hardy-weinberg-simulation

Environment Settings

This program has been successfully executed in the following environment.

Compiler: Microsoft Visual Studio 2013

OS: Windows 8 Enterprise, 64-bit
